# Association between body mass index and asthma severity in Arab pediatric population: A retrospective study

**DOI:** 10.1371/journal.pone.0226957

**Published:** 2019-12-27

**Authors:** Narjes Saheb Sharif-Askari, Hanan Abdulgader Sharif, Fatemeh Saheb Sharif-Askari, Qutayba Hamid, Salah Abusnana, Rifat Hamoudi

**Affiliations:** 1 Sharjah Institute for Medical Research, College of Medicine, University of Sharjah, Sharjah, United Arab Emirates; 2 College of Medicine, University of Sharjah, Sharjah, United Arab Emirates; Clinic for Infectious and tropical diseases, Clinical centre of Serbia, SERBIA

## Abstract

Increased body mass index (BMI) has been associated with an increased prevalence of asthma in children, however the association between BMI status and asthma severity has been less well defined. The aim of this study was to describe the association between childhood obesity and asthma severity, frequency of hospital and emergency department visits as well as pattern of aeroallergen sensitization. A retrospective study was conducted at pediatric outpatient clinics in University Hospital Sharjah. All consecutive patients aged 6 years and above, with confirmed diagnosis of asthma visiting the outpatient pediatric clinics during 2018 were included in this study. Sources of information were the patient’s medical file, laboratory data, pharmacy data, as well as reports from the pediatric in charge. This study included 164 children with asthma. 63% of asthma patients were male. The vast majority of patients were from Arab ethnicities (n = 154, 94%), majority had mild asthmatic conditions (n = 133, 81%), and one-third were either overweight or obese (n = 52, 32%). Overweight or obese asthmatic children with BMI percentile of equal or more than 85% was associated with more asthma severity (odds ratio [OR]: 3.27, 95% confidence interval [CI]: 1.42–7.54; *P =* 0.005), as well as more frequent asthma related hospital visits (OR: 2.53, 95% CI: 1.22–5.26; *P =* 0.013). Overweight asthmatic children with BMI between the 85^th^ and 94th percentiles and obese asthmatic children with BMI equal to or greater than 95th percentile are associated with more severe asthma phenotype and more frequent hospital and emergency department visits.

## Introduction

Childhood overweight and obesity is a preventable epidemic problem all over the world, a problem that has risen tenfold in the past four decades [1]. In 2010, 43 million children were estimated to be overweight and obese; 92 million were at risk of overweight [2]. Although the increase in obesity rate has plateaued in many developed countries, the prevalence of childhood obesity is accelerating in developing regions such as the Middle East and north Africa [1]. In United Arab Emirates, childhood obesity is a growing problem that has surpassed the international standards [3, 4]. Obesity is associated with chronic inflammation and metabolic dysregulation disturbing many of human systems such as immune, endocrine, cardiovascular and respiratory systems [5].

It has been proposed that obesity has role in development of asthma with obesity being associated with chronic central inflammation and asthma being associated with airway inflammation. According to world health organization, 235 million people currently suffer from asthma disease and obesity is one of the most common comorbidities of asthma [6].

Obese-asthma phenotype are difficult to treat and resistance to the conventional asthma treatments [7]. Additionally, uncontrolled asthma causes an increase in number of asthma exacerbation and emergency visits [8, 9].

Although previous studies, majority of which were conducted in United States, have reported higher prevalence of asthma among overweight and obese children, these studies have reported inconsistent data about severity and control of asthma among overweight and obese children [10–13]. From middle eastern country of Saudi Arabia, Nahhas et al. linked the increased BMI to higher asthma odds among boys and girls school children but they did not assess relationship between BMI and asthma severity [14]. In UAE, there is no previous study describing the link between obesity and asthma severity, while most of past asthma investigations provided general description of childhood asthma in the school setting using parental-reported questioners’ data which might be less accurate and more biased [15–17].

Therefore, this study was designed to be conducted in pediatric outpatient clinics. The main objective of this study was to determine the association between obesity and severity of asthma. The secondary objective was to assess the association of obesity with asthma exacerbation which was defined by frequency of asthma-related hospital and emergency department visit during the period of the study. Finally, the result for aeroallergen skin prick test was compared between asthmatic children with and without obesity.

## Methods

### Patients’ information and ethics

A retrospective study was conducted at pediatric outpatient clinics in University Hospital Sharjah. Ethical approval was obtained from the University Hospital Sharjah Research Ethics Board (Reference No: UHS-HERC- 053–13122018). In this study all patients’ records were analyzed in a fully anonymized and de-identified manner and no researcher had access to patients’ personal information, thus no consent was obtained.

### Inclusion and exclusion criteria

All consecutive patients between 6 and 18 years of age, with confirmed diagnosis of asthma visiting the outpatient pediatric clinics during 2018 were included in this study [18, 19].

A standard data collection form was designed for the purpose of this study. Sources of information were the patient’s medical file, laboratory data, pharmacy data, as well as reports from the pediatric in charge. Data was collected on sociodemographic, asthma characteristics, asthma medications, skin prick test results, and frequency of asthma-related hospital visits during the study period. Additionally, the severities of asthma included in this study: [1] intermittent, [2] mild persistent, [3] moderate persistent and [4] severe persistent were assessed and recorded routinely by physician following the National Asthma Education and Prevention Program [20].

Asthma medications were classified into the following categories: rescue medications alone or in combination (Salbutamol, Ipratropium), inhaled corticosteroids (Fluticasone, Budesonide), long beta-agonist (Salmeterol), oral steroids (Hydrocortisone, Dexamethasone, Prednisolone), anti-leukotriene-receptors (Montelukast).

Body mass index (BMI) percentiles were obtained from measured heights and weights using the Centers for Disease Control and Prevention (CDC) age and gender specific BMI growth charts and classified in to the following categories: children with a BMI percentile for age between 16% and 85% were classified as the normal BMI group, children with a BMI percentile for age of 85% to less than 95% were classified as overweight, and children with a BMI percentile for age of 95% or greater were classified as the obese group.

Skin prick test was conducted [21]. The following panel of 15 aeroallergens were tested: 2 mite species (Dermatophagoides farinae and Dermatophagoides pteronyssinus), 3 mold species (Alternaria alternata, Cladosporium mix containing Cladosporium cladosporioides and Cladosporium herbarum), Aspergillus mix (Aspergillus fumigatus, Aspergillus nidulans, Aspergillus niger), 2 weed species (Chenopodium album [fat hen] and Salsola kali [Russian thistle]), 2 grasses (Phleum pratense and Cynodon dactylon), tree pollen from date palm (Phoenix dactylifera), cat dander, feather mix (duck, goose, hen), rabbit and horse hair, and cockroach (Blattella germanica).

### Statistical analysis

In this study, categorical variables were reported as counts and percentages. Continuous variables were presented as mean ± standard deviation (SD) or median and interquartile range (IQR) if their distribution was skewed. Group comparisons were made by Student t test or Fisher exact test, as appropriate.

To determine the association of obesity with outcomes of asthma severity and frequency of hospital and emergency department visits a logistic regression analysis was adapted, which was adjusted for age and gender. All analyses were two-sided, with a P-value of <0.05 considered statistically significant. Analyses were undertaken using Statistical Package for Social Sciences (SPSS) version 25 (IBM Corp, New York).

## Results

During the study period, a total of 164 children with confirmed diagnosis of asthma visited the pediatric clinics. As shown in Table 1, study subjects were more likely to be males (63% male and 37% female; the median (IQ) age of these patient was 9 (4), range 6–18. Of these 164 children, vast majority (n = 154, 94%) were from Arab ethnicities, and majority of patients (133, 81%) had mild asthma condition with moderate to severe asthma contributing only to 19% of total cohort. Furthermore, out of 168 patients, the majority (n = 151, 92%) were prescribed Salbutamol, and more than one third (n = 60, 37%) were given Fluticasone medication.

**Table 1:** Socio-demographic and clinical characteristics of asthmatic children classified according to body mass index.

Variable	Total Asthma n = 164	BMI < 85^th^ percentile asthmatics n = 112	BMI ≥ 85^th^ percentile asthmatics n = 52	*P*-value
Age (years), median (IQR)	9 (4)	9 (4)	8.5 (4)	0.182
Gender				0.729
Male, n (%)	103 (63)	69 (62)	34 (65)	
Female, n (%)	61 (37)	43 (38)	18 (35)	
BMI (kg/m2) percentile), median (IQR)	62 (56)	47 (46)	96 (7)	<0.001
Asthma severity				0.002
Intermittent-mild asthma	133 (81)	98 (88)	35 (67)	
Moderate-severe asthma	31 (19)	14 (12)	17 (33)	
Nationality		3 (3%)	3 (6)	0.720
UAE national, n (%)	113 (69)	75 (67)	38 (73)	
Other Arabs, n (%)	41 (25)	30 (27)	11 (21)	
Others, n (%)	10 (6)	7 (6)	3 (6)	
Serum Eosinophil (count/mm^3^), median (IQR)	170 (470)	170 (470)	190 (590)	0.392
Serum Neutrophil (%), median (IQR)	57.7 (28)	57.2 (29)	61.5 (28)	0.731
Salbutamol, n (%)	151 (92)	101 (90)	50 (96)	0.230
Ipratropium, n (%)	41 (25)	26 (23)	15 (29)	0.551
Salmeterol, n (%)	21 (13)	9 (8)	12 (23)	0.011
Budesonide, n (%)	3 (2)	2 (2)	1 (2)	0.951
Fluticasone, n (%)	60 (37)	39 (35)	21 (40)	0.492
Mometasone, n (%)	16 (10)	11 (10)	5 (10)	0.901
Montelukast, n (%)	33 (20)	26 (23)	7 (13)	0.190
Atopic positive*	56 (34)	41 (37)	15 (29)	0.379
Skin Prick test				
House dust mite, n (%)	42 (58)	31 (61)	11 (50)	0.392
Molds, n (%)	35 (48)	29 (57)	6 (27)	0.020
Grass mix, n (%)	23 (32)	20 (39)	3 (14)	0.031
Weed, n (%)	18 (25)	15 (29)	3 (14)	0.151
No. of hospital visits ≥2, n (%)	47	26 (23)	21 (40)	0.027

* Atopic status was determined using the skin prick test and it was available for 73 (45%)

The distribution of BMI percentile classes was as following: 13 (8%) were underweight, 99 (60%) had healthy weight, 21 (13%) were overweight, and 31 (19%) were obese. The underweight group were aged between 6 and 12 years, the healthy weight group were aged between 6 and 18 years and overweigh and obese group were aged between 6 and 12 years. Compared with healthy weight asthmatic children, overweight or obese individuals had more moderate-severe asthma (12% vs. 33%) and this difference was statically significant (*P* = 0.002). Additionally, Salmeterol medication was more commonly prescribed in obese asthmatic children (8% vs. 23%; *P =* 0.011).

Skin prick test was performed for 73 (45%) of patients, and three-fourth of these test were positive (n = 56, 34%). The 13 aeroallergen types were categorized in to four groups of house dust mite, weed mix, grass mix, and mold. Based on skin prick test, around half of asthmatic patients were sensitized to house dust mite (n = 42, 58%), and molds (n = 35, 48%). And, 10 out of 56 (18%) atopic patients were positive to more than 2 aeroallergens. The was no statistically significant difference in overall sensitization between the healthy weight and overweight or obese asthmatic children (37% vs 29%, *P* = 0.379). However, compared to healthy weight atopic asthma patients, overweight or obese atopic subjects were less sensitized to aeroallergen groups of molds (57% vs 27%, *P* = 0.020) and grass mix (39% vs 14%, *P* = 0.031). The sensitization variables were not entered into the logistic regression model because the test was only performed in 73 (45%) of patients.

Overweight or obese asthmatic children with BMI percentile of equal or more than 85% were associated with more asthma severity (OR: 3.27, 95% CI: 1.42–7.54; *P =* 0.005), as well as more frequent asthma related hospital visits (OR: 2.53, 95% CI: 1.22–5.26; *P =* 0.013). Table 2 displays comparison of asthma outcomes between healthy weight and overweight or obese children with asthma.

**Table 2:** Comparison of asthma outcomes between healthy weight and overweight or obese children with asthma.

	Crude OR (95% CI)	Adjusted OR (95% CI) *	*P*-Value
**Moderate-severe asthma**	3.4 (1.52–7.61)	3.27 (1.42–7.54)	0.005
**No. of hospital visits ≥2**	1.13 (1.07–1.17)	2.53 (1.22–5.26)	0.013

CI, confidence interval; OR, odds ratio.

*Adjusted for age and gender

## Discussion

Results from this study that was conducted among Arab pediatric population suggest that higher BMI contributed to more severe asthma phenotype and more frequent hospital and emergency department visits. The aeroallergen skin pricks univariate analysis showed that compared to healthy weight children with asthma, there was no statistically significant difference in overall sensitization between overweight or obese and healthy weight patients.

More than half of asthma patients were male, one-third were either overweight or obese, and majority had mild asthma. In agreement with previous studies, asthma was more prevalent in boys than in girls [22, 23], but percentages of boys and girls did not differ between overweight or obese and healthy weight asthmatic children. Moreover, one-third of asthmatic children were either overweight or obese. These results were fairly consistent with previous studies [19].

In adult, obesity is associated with more severe asthma and a poorer asthma control [24, 25]. Childhood obesity has been linked with an increased incidence of asthma in children [15, 16], however inconsistent association between childhood obesity and asthma severity have been reported via previous investigations [8, 17, 18]. While some studies such as Black et al [26] and Stingone et al [27] reported significant association between obesity and rate of hospital and emergency department visits, others did not. Peters et al [28] reported no link between BMI and hospital and emergency department visits. Quinto and colleagues [19] reported that although childhood obesity was associated with increased number of beta against and oral corticosteroid intake, there was no association between obesity and rate of hospital visits [19]. In our study, childhood obesity was associated with more severe asthma phenotype and more frequent hospital and emergency department visits.

Although a number of previous studies have reported positive association between higher BMI and overall sensitization [29, 30], the rest majority and the current study could not find significant association [31]. Looking at individual aeroallergen’s groups, compared to healthy weight children with asthma, overweight or obese individuals were less sensitized to aeroallergen groups of molds and grass mix, whereas sensitization to house dust mite did not differ between obese and non-obese patients. Our sensitization data was derived from univariate analysis and was not entered into logistic regression because the atopy test was not performed in more than half of patients. Beside the current study, few studies that have described the link between higher BMI and individual aeroallergen sensitization have reported conflicting results. Van Gysel et al. [31] found no difference in overall and specific aeroallergen sensitization between non-obese and obese children with asthma. Forno et al. reported that BMI, percent of body fat, and waist circumference each consistently were associated with higher sensitization to aeroallergens of cockroach, mold, and mouse, whereas sensitization to house dust mite did not differ between obese and non-obese patients [30].

The reasons behind inconsistent association between obesity and asthma severity and atopy could be attributed to a number of reasons. Different studies have used variable population, different age range, different definition of asthma diagnosis, severity and outcomes. Furthermore, study design and settings also varied such as cohort studies conducted in hospital settings or a survey conducted among children and their parents in school settings. Finally, inconsistencies among investigations, including ours, may be explained by differences in social, environmental, and genetic factors.

The mechanisms by which obesity adversely influences asthma prevalence, severity, outcomes and atopy are not well understood, and further information needed about whether obesity comes first and cause asthma development or it comes later as consequence of limited physical activity in asthmatic children. It is proposed that obesity affect asthma through altered lung structure, decrease in nitric oxide levels and increase in levels of systematic inflammation, adipocytokines and oxidative stress [32]. As atopy often begins in childhood it may be that higher BMI in childhood is important for the development of atopy or sensitization to a specific aeroallergen groups as in case of current study[33]. The relationship between BMI and atopy among children with asthma should be further explored.

The findings of the present study may be valuable for public health authorities as it provides additional evidence of considerable worse asthma status associated with presence of a modifiable risk factor of obesity. Weight reduction in asthmatic children with high BMI could help to improve their asthma control, medication use and hence reduce the rate of asthma exacerbation and the need for hospital and emergency department visits.

In summary, overweight BMI between the 85^th^ and 94^th^ percentiles and obese BMI equal to or greater than 95^th^ percentile were both linked to more severe asthma and more frequent hospital visits. Although overall atopy was not different between non-obese and obese asthmatic children, in univariate analysis, the examination of individual aeroallergen sensitization revealed that obese and overweight children were less sensitized to aeroallergen groups of molds and grass mix. These sensitization results need to be confirmed via larger cohort of patients.

## Supporting information

S1 Data (SAV)
